# A key to known species of *Episcapha* (subgenus *Ephicaspa* Chûjô) (Coleoptera, Erotylidae, Megalodacnini), with the description of two new species

**DOI:** 10.3897/zookeys.203.3400

**Published:** 2012-06-20

**Authors:** Jing Li, Guo-Dong Ren

**Affiliations:** 1 College of Plant Protection, Agricultural University of Hebei, Baoding, Hebei, 071002, P. R. China; 2 College of Life Sciences, Hebei University, Baoding, Hebei 071002, P. R. China

**Keywords:** Coleoptera, Erotylidae, Megalodacnini, *Episcapha*, *Episcapha*, key, new species

## Abstract

Two new species *Episcapha (Ephicaspa) lushuiensis* **sp. n.** and *Episcapha (Ephicaspa) quadriconcava* **sp. n.** are described and illustrated from China. A key to known species of subgenus *Ephicaspa* is provided. A map of the collecting sites is given.

## Introduction

To date, 3 subgenera of the genus *Episcapha* Lacordaire, 1842 have been known (Lacordaire 1842; Heller 1918; Chûjô 1969). Among them, subgenus *Ephicaspa* was elected by Chûjô for *Episcapha asahinai* Chûjô, 1936 as the type species. The subgenus *Ephicaspa* only included 3 species worldwide. *Episcapha (Ephicaspa) asahinai* Chûjô was described from China (Taiwan) to Japan (Chûjô 1936; Araki 1941; Chûjô 1969). *Episcapha (Ephicaspa) lewisi* Nakane was reported from Japan (Nakane 1950; Nakane 1955). *Episcapha (Ephicaspa) yunnanensis* Li & Ren was described from Yunnan Province, China (Li and Ren 2006).

In the current work, two new species of the subgenus *Ephicaspa* are described and illustrated. One new species, *Episcapha (Ephicaspa) lushuiensis* sp. n., was collected from Yunnan Province, China. The other new species, *Episcapha (Ephicaspa) quadriconcava* sp. n., was collected from Guangxi Zhuang Autonomous Region, China. A key to species of subgenus *Ephicaspa* is provided. A map (Fig. 23) of the collecting sites is given.

## Methods

The specimens were collected by chopping deadwood in forests. They were killed with ethyl acetate and dried. The morphological examinations were carried out with a stereomicroscope. For an examination of the male or female genitalia, the last three abdominal segments were detached from the body after softening in hot water. For clearing, it was boiled for 5 minutes in 5% solution of potassium hydroxide, and then, washed in distilled water. Morphological figures were prepared using a Nikon SMZ1500 stereomicroscope. All measurements are given in millimetres. The habitus photos were taken with a Leica M205A camera. Holotypes and paratypes are deposited in the Museum of Hebei University (MHU), Hebei, P. R. China.

Morphological terminology predominantly follows Wegrzynowicz (1997) with changes according to Skelley and Leschen (2007).

The measurements of proportions are abbreviated as follows:

bl/bw – body length/width ratio;

pl/pw – pronotum length/width ratio.

## Descriptions

### Episcapha (Ephicaspa) lushuiensis sp. n.

urn:lsid:zoobank.org:act:DFA4FEBC-E3BC-4C29-945C-D500E8093F0B

http://species-id.net/wiki/Episcapha_lushuiensis

#### Type material.

Holotype. male, CHINA: Yunnan Province, Lushui County, 25.9667°N, 98.8167°E, 11 May 2004, Zi-Zhong. YANG leg (MHU). Paratypes. 2 males, 8 females, same data as holotype (MHU).

#### Description.

**Figures 1–11.**
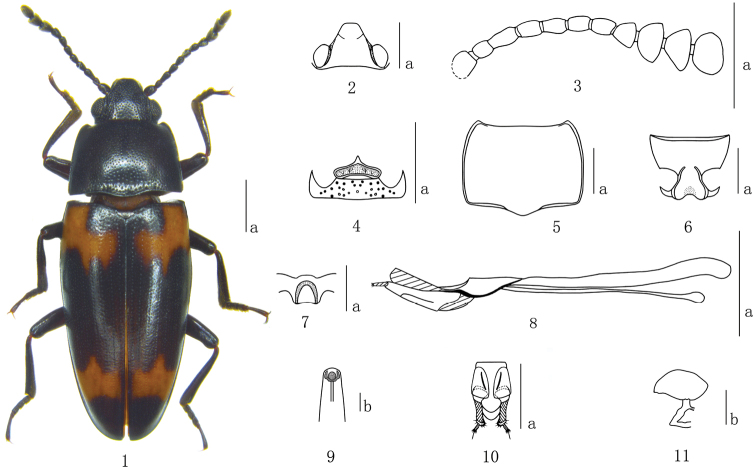
*Episcapha (Ephicaspa) lushuiensis* sp. n. **1** habitus **2** head **3** antenna **4** mentum **5** Pronotum **6** prosternum **7** mesoventrite **8** aedeagus in lateral views **9** anterior end of internal sac in anterodorsal view **10** female genitalia in ventral view **11** female spermatheca Scale bars: **a** = 1mm, **b** = 0.1mm.

Body (Fig. 1) strongly elongate, length: 6.5–8.0mm; width: 2.3–3.0mm (bl/bw = 2.59–2.72; average = 2.63); general color black, moderately shining; mouthparts and tarsi brown to reddish brown. Each elytron with 2 orange bands; anterior band extending obliquely from humerus to near the suture, abruptly narrowed in middle, with 3 teeth on anterior border, reaching the base at the humeral angle; posterior band at four fifths length of elytron, extending from the suture to near the lateral border, with 3 teeth at anterior border, with posterior border slightly curved.

Head (Fig. 2) strongly and sparsely punctured on vertex, with ocular lines. Clypeus finely and closely punctured, with anterior border nearly straight, with a fovea on each side of the base. Eyes large, moderately prominent laterally. Antennae (Fig. 3) long, extending a little behind posterior border of pronotum; antennomere III about 1.3 times as long as IV; antennomere VIII slightly wider than VII, about 1.2 times as wide as long; antennomeres IX hemispherical; antennomere X almost asymmetrical triangular; antennomere XI roundly quadrate, slightly constricted in middle; relative lengths of antennomeres II–XI: 8.0: 14.5: 11.5: 12.0: 11.0: 12.0: 10.0: 12.0: 12.0: 15.0. Mentum (Fig. 4) pentagonal, pointed apically, with coarse punctures and setae; submentum roundly and roughly punctured.

Pronotum (Fig. 5) widest at base (pl/pw = 0.72–0.80; average = 0.75); sides almost parallel on posterior half, and slightly narrowing toward apex. Pronotum distinctly punctured on median area, slightly decreasing in size and increasing in density toward lateral areas. Anterior angles roundly projected; posterior angles nearly rectangulate. Prosternum (Fig. 6) strongly and rather closely punctured on the lateral areas, strongly and sparsely punctured on median area, with a longitudinal depression in the middle of base area. Prosternal process dilated apically; strongly emarginated at apical border. prosternal femoral lines convergent anteriorly.

Scutellum broadly pentagonal, finely and sparely punctured.

Elytra widest near base, then gradually narrowing to apex; each elytron with 8 striae; intervals with fine and sparse punctures, which are much finer than those in striae.

Mesoventrites (Fig. 7) finely and sparsely punctured, with an n-shaped depression medially.

Aedeagus (Fig. 8) slightly curved; median lobe narrow, with apex truncate in lateral view; median strut long, about 2.0 times as long as median lobe. Anterior end of internal sac as in Fig. 9.

Female genitalia (Fig. 10) with styli most narrow at base; proctigeral lobe acuminate apically; female spermatheca (Fig. 11) with head almost spindle shaped.

#### Distribution.

Known only from the type locality (China: Yunnan Province, Lushui County).

#### Diagnosis.

This new species is closest to *Episcapha (Ephicaspa) asahinai* Chûjô, 1936 due to similar form and color of the body. The new species can be distinguished from *Episcapha (Ephicaspa) asahinai* by antennomere III more than 1.8 times as long as II, prosternal femoral lines convergent anteriorly, body beneath with golden pubescence. *Episcapha (Ephicaspa) asahinai* with antennomere III about 1.5 times as long as II, prosternal femoral lines almost straight and parallel in front of the prosternal cavity, body beneath covered with grayish pubescence.

#### Etymology.

The specific name derives from the type locality: Lushui County.

### Episcapha (Ephicaspa) quadriconcava sp. n.

urn:lsid:zoobank.org:act:A1331079-C03F-4211-B836-94E12041B86E

http://species-id.net/wiki/Episcapha_quadriconcava

#### Type material. 

Holotype. female, CHINA: Guangxi Zhuang Autonomous Region, Leye County, 24.7833°N, 106.5666°E, 26 VII 2004, Yang YU and Chao GAO (MHU). Paratypes. 1 male and 4 females, same data as holotype (MHU).

#### Description.

**Figures 12–22.**
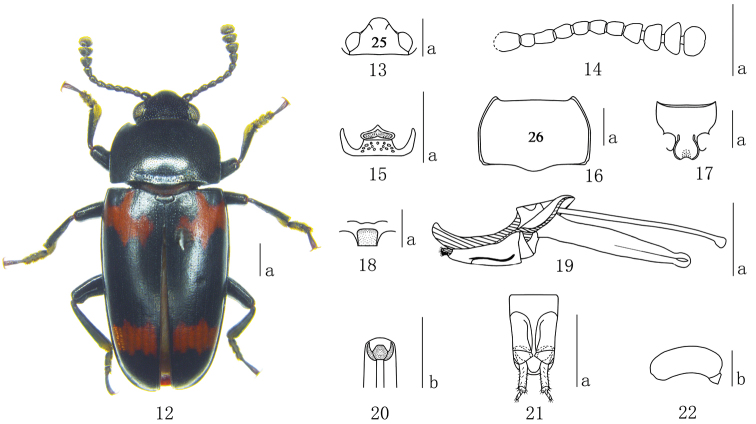
*Episcapha (Ephicaspa) quadriconcava* sp. n. **12** habitus **13** head **14** antenna **15** Mentum **16** pronotum **17** prosternum **18** mesoventrite **19** aedeagus in lateral views **20** anterior end of internal sac in anterodorsal view **21** female genitalia in ventral view **22** female spermatheca Scale bars: **a** = 1mm, **b** = 0.2mm.

Body (Fig. 12) elongate, length: 8.5–9.1 mm; width: 3.3–3.6 mm (bl/bw = 2.53–2.58; average = 2.56); general color black and shining; mouthparts and tarsi brown. Each elytron with 2 orange bands; anterior band extending obliquely from humerus to the middle of striae I and II, with 3 teeth at anterior and posterior borders, not reaching base at humeral angle; posterior band at four fifths length of elytron, almost quadrate, extending from the suture to near the lateral border.

Head (Fig. 13) strongly and sparsely punctured on vertex, without ocular lines. Clypeus finely and closely punctured, with anterior border nearly straight, with a fovea on each side of the base. Eyes large, prominent laterally. Antennae (Fig. 14) long, extending behind posterior border of pronotum; antennomere III about 1.2 times as long as IV; antennomere VIII slightly wider than VII, about 1.2 times as wide as long; antennomere IX blow-shaped; antennomere X almost asymmetrical triangular; antennomere XI almost pentagonal; relative lengths of antennomeres II–XI: 8.0: 12.0: 10.0: 9.0: 9.0: 10.0: 10.0: 11.0: 10.0: 13.0. Mentum (Fig. 15) pentagonal, pointed apically, with fine punctures and short setae; submentum with sparse coarse punctures.

Pronotum (Fig. 16) widest at middle (pl/pw = 0.59–0.63; average = 0.61); sides almost parallel on posterior half, and narrowing toward apex. Pronotum distinctly punctured on median area; finely and closely punctured on the lateral areas; with a transverse depression at base. Anterior angles projected; posterior angles obtuse. Prosternum (Fig. 17) coarsely and densely punctured on lateral areas, almost confluent and forming oblique rugae; surface with golden pubescence. Prosternal process dilated apically; strongly emarginated at apical border; distinctly depressed in the middle. Prosternal femoral lines extended a little in front of the prosternal cavity, almost straight and parallel.

Scutellum pentagonal, with fine and spare punctures.

Elytra widest at one fourth from base, then gradually narrowing to apex; each elytron with 7 striae; intervals with fine punctures.

Mesoventrite (Fig. 18) finely and sparsely punctured, with a median quadrate depression; surface with pubescence.

Aedeagus (Fig. 19) with median lobe moderately curved, narrowing to a point in lateral view; median strut long, about 1.5 times as long as median lobe. Anterior end of internal sac as in Fig. 20.

Female genitalia (Fig. 21) with styli most narrow at apex; proctigeral lobes rounded apically; female spermatheca (Fig. 22) with head almost kidney-shaped.

#### Distribution.

Known only from the type locality (China: Guangxi Zhuang Autonomous Region, Leye County).

**Figure 23.**
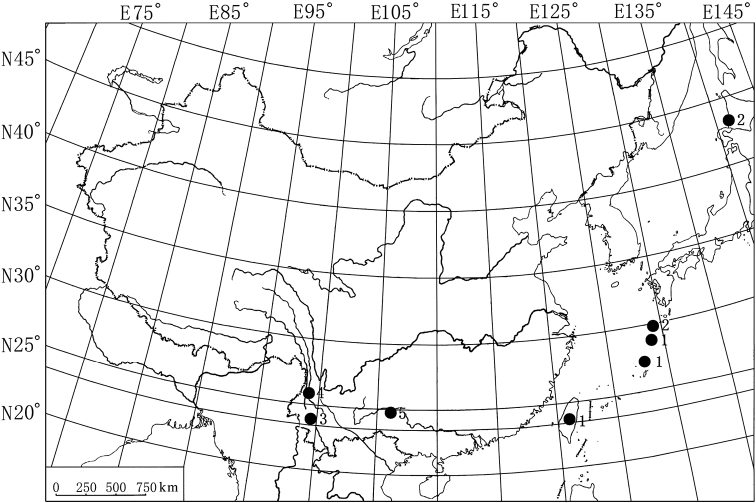
Map showing the collecting sites of the *Episcapha* (*Ephicaspa*) Chûjô. **1** *Episcapha (Ephicaspa) asahinai* Chûjô; **2** *Episcapha (Ephicaspa) lewisi* Nakane; **3** *Episcapha (Ephicaspa) yunnanensis* Li & Ren; **4** *Episcapha (Ephicaspa) lushuiensis* sp. n.; **5** *Episcapha (Ephicaspa) quadriconcava* sp. n.

#### Diagnosis.

*Episcapha (Ephicaspa) quadriconcava* is closest to *Episcapha (Ephicaspa) yunnanensis* Li & Ren, 2006, due to the similar form and color of the body in both species. The new species can be distinguished from *Episcapha (Ephicaspa) yunnanensis* by the head without ocular lines, pronotum widest at middle, mesoventrite with a median quadrate depression, aedeagus with median lobe narrowing to a point in lateral view. *Episcapha (Ephicaspa) yunnanensis* with ocular lines on head, pronotum widest at base, mesoventrite with a median trapezoidal depression, aedeagus with median lobe hooked at apex in lateral view.

#### Etymology.

The species is named with a quadrate depression on the median of mesoventrite.

##### Key to the species of subgenus *Ephicaspa* Chûjô

**Table:** 

1	Elytron with the anterior band wholly occupying the latero-basal area including humerus	2
–	Elytron with the anterior band extending towards the basal border at each side of humerus	3
2	Prosternal femoral lines almost straight and parallel in front of the prosternal cavity	*Episcapha (Ephicaspa) asahinai* Chûjô
–	Prosternal femoral lines convergent anteriorly in front of the prosternal cavity (Fig. 6)	*Episcapha (Ephicaspa) lushuiensis* sp. n.
3	Scutellum broad triangular	*Episcapha (Ephicaspa) lewisi* Nakane
–	Scutellum broad pentagonal	4
4	Mesoventrites with a median obtrapeziform depression; aedeagus with median lobe hooked at apex in lateral view	*Episcapha (Ephicaspa) yunnanensis* Li & Ren
–	Mesoventrite with a median quadrate depression (Fig. 18); aedeagus with median lobe narrowing to a point in lateral view (Fig. 19)	*Episcapha (Ephicaspa) quadriconcava* sp. n.

## Supplementary Material

XML Treatment for Episcapha (Ephicaspa) lushuiensis

XML Treatment for Episcapha (Ephicaspa) quadriconcava
